# Effects of measurement methods and growing conditions on phenotypic expression of photosynthesis in seven diverse rice genotypes

**DOI:** 10.3389/fpls.2023.1106672

**Published:** 2023-09-21

**Authors:** Megan Reavis, Larry C. Purcell, Andy Pereira, Kusum Naithani

**Affiliations:** ^1^ Department of Biological Sciences, University of Arkansas, Fayetteville, AR, United States; ^2^ Department of Crop, Soil, and Environmental Sciences, University of Arkansas, Fayetteville, AR, United States

**Keywords:** genotypes, light response curve, phenotypic plasticity, phenotyping, photosynthesis model, rice, sequential and non-sequential method

## Abstract

**Introduction:**

Light response curves are widely used to quantify phenotypic expression of photosynthesis by measuring a single sample and sequentially altering light intensity within a chamber (sequential method) or by measuring different samples that are each acclimated to a different light level (non-sequential method). Both methods are often conducted in controlled environments to achieve steady-state results, and neither method involves equilibrating the entire plant to the specific light level.

**Methods:**

Here, we compare sequential and non-sequential methods in controlled (greenhouse), semi-controlled (plant grown in growth chamber and acclimated to field conditions 2-3 days before measurements), and field environments. We selected seven diverse rice genotypes (five genotypes from the USDA rice minicore collection: 310588, 310723, 311644, 311677, 311795; and 2 additional genotypes: Nagina 22 and Zhe 733) to understand (1) the limitations of different methods, and (2) phenotypic plasticity of photosynthesis in rice grown under different environments.

**Results:**

Our results show that the non-sequential method was time-efficient and captured more variability of field conditions than the sequential method, but the model parameters were generally similar between two methods except the maximum photosynthesis rate (*A_max_*). *A_max_* was significantly lower across all genotypes under greenhouse conditions compared to the growth chamber and field conditions consistent with prior work, but surprisingly the apparent quantum yield (α) and the mitochondrial respiration (*R_d_*) were generally not different among growing environments or measurement methods.

**Discussion:**

Our results suggest that field conditions are best suited to quantify phenotypic differences across different genotypes and nonsequential method was better at capturing the variability in photosynthesis.

## 1 Introduction

Expected global temperature rise and water scarcity (IPCC, 2018) present serious threats to crop production and global food security. To feed the growing human population without using more land while reducing water use and greenhouse gas emissions, we need to investigate the limitation of commonly used measurement and modeling techniques used for quantifying gene and environment interactions. Photosynthesis response (to light, CO_2_, and temperature) curves are commonly used to estimate species-specific parameters, including maximum photosynthetic capacity, maximum electron transport rate, mitochondrial respiration, maximum light use efficiency, maximum rate of photosynthesis, and optimal temperature (Berry and Bjorkman, 1980; Farquhar et al., 1980; Battaglia et al., 1996; Medlyn et al., 2002; Murchie et al., 2002; Ralph and Gademann, 2005; Lobo et al., 2013). These parameters provide insight into the intrinsic characteristics of the plants based on biological mechanisms. Understanding crop response to variable environmental conditions is critical to improve our predictions of crop yield that are often correlated with the plant biomass and photosynthesis (Zelitch, 1982; Bouman and Tuong, 2001; Gu et al., 2013). To meet the increasing demand of food, the global human population is expected to reach 9 billion by 2050, and adapt to a warming planet (global temperatures are expected to rise more than 2°C by 2050 without deep emission reductions; IPCC, 2018), predictive crop models based on mechanistic understanding of photosynthesis response to changing environmental conditions are needed for selecting and breeding plants for desirable attributes.

Conventionally, photosynthesis light response curves are generated by clamping one or more leaves into a chamber and sequentially altering the light intensity within the chamber of a gas exchange measurement system (McDermitt et al., 1989; Ögren, 1993; Dreyer et al., 2001). This method requires significant time for each curve, as plants need to acclimate for several minutes at each light level (Battaglia et al., 1996; Serôdio et al., 2013). One example of a sequential light response curve is the steady-state light response curve, which requires 10-20 minutes at each light level to allow the plant to acclimatize to the current light level; this allows for characterization of the plasticity and inherent steady-state photosynthesis properties at different light intensities (Coe and Lin, 2018). Another example of a sequential light response curve is a rapid light response curve that can be generated relatively quickly, with only 1-3 minutes needed at each light level (Coe and Lin, 2018; LI-COR, 2021). They can also be used to characterize a plant’s dynamic photosynthetic response under rapidly fluctuating light conditions (Ralph and Gademann, 2005; Coe and Lin, 2018). Non-sequential (or survey) light response curves are similar to rapid sequential light response curves, but rather than subjecting the same sample to a sequence of light intensities, different samples equilibrated at different light intensities are used to build a similar curve (Perkins et al., 2006; Houliez et al., 2017; Coe and Lin, 2018; LI-COR, 2021). Non-sequential light response curves are often conducted on microalgae (Perkins et al., 2006), phytoplankton (Houliez et al., 2017), or other marine plants (Ralph and Gademann, 2005) using chlorophyll fluorescence parameter response to light rather than net photosynthesis.

In each of these methods (sequential and non-sequential) for generating light response curves in land plants, a portion of a leaf is enclosed in an artificial environment, different from the rest of the plant. This difference between the whole plant and measuring environment may limit the reliability of the conventional response curves to estimate the full photosynthetic capacity of the whole plant (Wagner and Reicosky, 1992; Sims et al., 1998). Additionally, most plants are measured in controlled environments, but recent studies demonstrating phenotypic plasticity of plants (Sultan, 2000; Pieruschka and Schurr, 2019) suggest that plant response under controlled environmental conditions will likely differ from their response under field conditions (Sultan, 2000). Rice photosynthetic properties are widely studied in controlled (e.g., Xu et al., 2019; Lv et al., 2020) and field (e.g., Murchie et al., 2002) conditions using light response curves, but none of the prior studies have used a non-sequential method (i.e., using different samples acclimated at different light levels) under field conditions. If non-sequential light response curves are conducted in a field environment, the whole plant is equilibrated to the same environmental conditions as the sample being measured (e.g., light, temperature, humidity). This type of curve eliminates the effect of the previous light intensities on the current measurement (Coe and Lin, 2018) and the measurements are taken on the samples acclimated to the surrounding environmental conditions (Coe and Lin, 2018; LI-COR, 2021). Here, we compared the phenotypic expression of photosynthesis using sequential and non-sequential light response curves in seven rice (*Oryza sativa* L.) genotypes grown under different environmental conditions to understand phenotypic plasticity of plants and limitations of different measurement methods.

To understand the effects of measurement methods and growing environments on photosynthetic traits across seven rice genotypes, we estimated photosynthesis model parameters and associated uncertainties across different growing environments and measurement methods by implementing a widely used non-rectangular hyperbola model (Thornley and Johnson, 1990) in a multilevel Bayesian framework. First, we asked whether the phenotypic expression of photosynthesis, estimated by photosynthesis model parameters, differs depending on the measurement methods employed? We expect the similar results across different measurement methods as both methods are frequently used to quantify photosynthesis response to light. Second, we investigated the differences in phenotypic expression of photosynthetic traits between plants grown under controlled and field conditions. Recent studies suggest that plant response under controlled environmental conditions will likely differ from their response under field conditions (Sultan, 2000; Pieruschka and Schurr, 2019). We expect to see significant differences in photosynthesis traits across growing conditions.

## 2 Methods

### 2.1 Plant material

Five out of the seven genotypes used in this research were selected from the USDA rice mini-core collection (310588, 310723, 311644, 311677, 311795), a collection of 217 genotypes with diverse origins, subgroups, and phenotypic and genotypic characteristics (Agrama et al., 2009; Kumar, 2017). Two additional genotypes outside of the USDA rice mini-core collection selected for this study were a drought tolerant *aus* genotype Nagina 22 (N22) and a drought sensitive *indica* genotype Zhe733. Details of each of these genotypes are provided in **Table 1**.

**Table 1:** Information about rice (Taxon = *Oryza sativa*) genotypes and different growing environments: F, field; GH, greenhouse; GC, growth chamber plants acclimated to field conditions.

USDA Accession No.	Genotype Name	Subgroup	Country of Origin	Growing Environment
310723	WIR 3039	AUS	Tajikistan	F, GC
311644	P 35	AUS	India	F, GC
311677	Karabaschak	TEJ	Bulgaria	F, GC
—	Zhe 733	IND	China	F, GC
310588	Onu B	TRJ	Zaire	F, GC, GH
311795	Nipponbare	TEJ	Japan	F, GC, GH
—	Nagina 22	AUS	India	F, GC, GH

### 2.2 Photosynthesis light response curve measurements

#### 2.2.1 Field experiment

Five replicates of each genotype were germinated in pots and transplanted (21 day after germination) to a 6 x 6 m levee-bound plot at the University of Arkansas Division of Agriculture’s Agriculture Experiment Station in Fayetteville, AR (36.096051°, -94.167418°). Plants were transplanted 0.3 m apart from each other in rows by genotypes with five replicates per genotype, a total of 35 plants. A flood of 2-10 cm was maintained in the field for the entire growing season of 2019. The field soils are a combination of Pembroke silt loam (fine-silty, mixed, active, mesic Mollic Paleudalfs) and Pickwick wilt loam (fine-silty, mixed, semi-active, thermic Typic Paleudults) (USDA Web Soil Survey, 2021).

**Figure 1:**
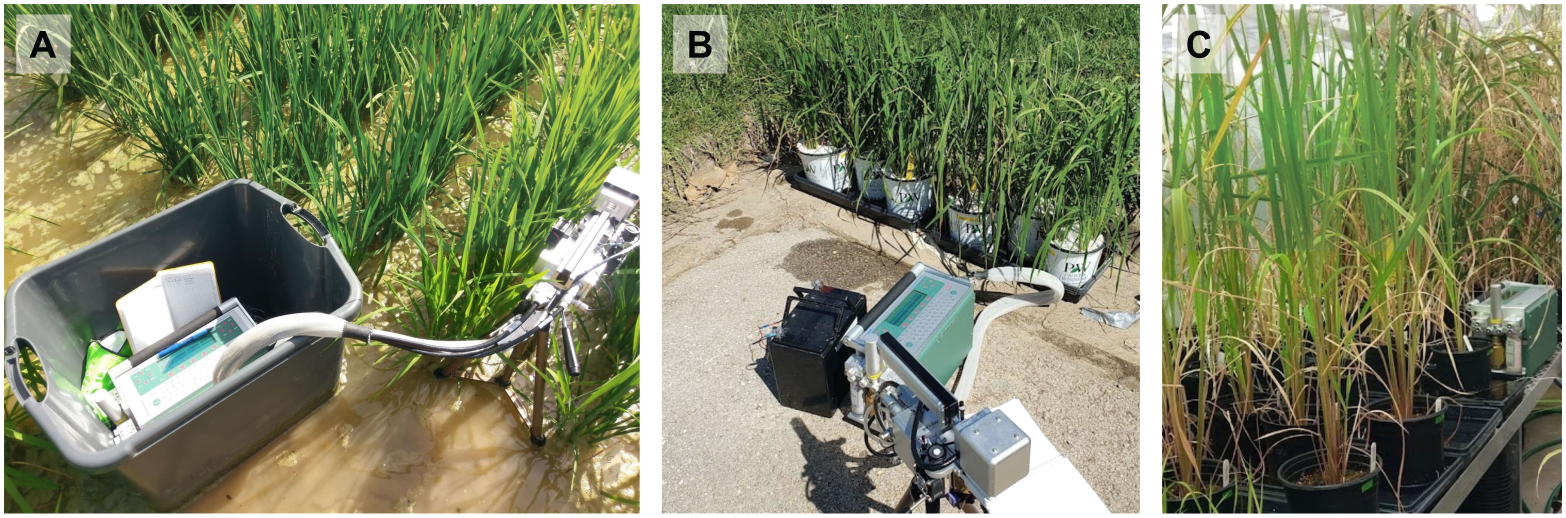
Images showing measurements of rice plants with the LI-6400XT across different growing environments including **(A)** field, **(B)** growth chamber-grown pots acclimated to field conditions, and **(C)** greenhouse conditions.

Light response curves were generated *in situ* for plants grown in the field (**Figure 1A**) using the non-sequential survey method (Perkins et al., 2006; Houliez et al., 2017; Coe and Lin, 2018; LI-COR, 2021) where diurnal light intensities and multiple plant replicates were used to fully capture the variability and photosynthetic range within each genotype. These curves take advantage of the natural diurnal light pattern of the day and the photosynthetic response of the whole plant to those changing light levels to create the curve rather than altering the environment of a small portion of a leaf. For these curves, instantaneous measurements with the LI-6400XT (LI COR Biosciences, Lincoln, NE, USA) began around 0700 h and were collected throughout the day on recently matured healthy, unshaded leaves of the canopy on all replicates until 1700 h, or until three measurements on each plant were taken. Light intensities can vary greatly within the rice canopy (Burgess et al., 2017), so we ensured that the leaves in all experiments were unshaded and acclimated to the ambient, full-light conditions. A complete curve for a genotype had 15 data points (*three measurements x five replicates*), which is comparable to a typical, sequential light response curve. Photosynthetically active radiation (PAR) and air temperature within the leaf chamber was set to reflect ambient PAR (varied from 1 to 2400 *µ*mol photon m^-2^ s^-1^), temperature (varied from 24 to 36°C), and vapor pressure deficit (VPD, varied from 0.79 to 3.5 kPa). Proper care was taken to ensure the sensor was not shaded by leaves or the researchers.

#### 2.2.2 Growth chamber experiment

Growth chamber plants were germinated in a greenhouse, three seeds to a 3.8 L pot, then moved to a growth chamber after 4 weeks. Soil medium was 5:1 potting soil to field soil. Lights in the growth chamber were set to the maximum light intensity (600 *µ*mol m^-2^ s-^1^) on a 14-10 light-dark cycle and temperature was set to 28 °C. Pots were watered daily to ensure adequate soil moisture.

Growth chamber response curves were generated using the sequential response curve methodology for comparison with the non-sequential response curves of field plants as both growth chamber and field plants were acclimated to the field environmental conditions before taking the measurements. After growing for 3 weeks in the growth chamber, pots were moved outside into water-filled trays in the sunlight where they were allowed to acclimate for 2-3 days (**Figure 1B**). An auto-program (reference CO_2_ set to 410 ppm, block temperature and VDP set to ambient (varied, 26-40°C and 1-4 kPa)) with 11 light levels (2000, 1600, 1400, 1200, 1000, 800, 600, 400, 200, 50 *µ*mol m^-2^ s^-1^) was then run on recently matured, unshaded healthy leaves of three plants of each genotype. The experiment was repeated for a total of three rounds, however, seeds for N22 only germinated for one round, resulting in fewer replicates. Air temperature during measurements ranged from 24-38 °C, with an average temperature of 33 °C. Relative humidity was around 47%, and PAR ranged between 1300-2300 *µ*mol m^-2^ s^-1^ during measurements, taken between 1100 and 1400 h.

#### 2.2.3 Greenhouse experiment

Greenhouse studies were conducted in University of Arkansas’s greenhouses in Fayetteville, AR (**Figure 1C**). Plants were grown in 0.95 L pots in trays of water to simulate flooded conditions. Pots were filled with mixed soil (5:1 potting soil to autoclaved field soil). Field soil was collected from the same location where the field experiment was conducted. We used the sequential, rapid method of measuring photosynthesis response to light with the LI-6400XT on recently matured, unshaded healthy leaves. An auto-program was created to collect data at 10 light levels (1400, 1200, 1000, 800, 600, 400, 200, 100, 50, 0 *µ*mol m^-2^ s^-1^) beginning at the highest light and allowing up to 3 minutes of acclimation at each level. During measurements air temperature, VPD, and PAR ranged from 24.9-26.4 °C, 1.0-2.7 kPa, and 1300-2300 *µ*mol m^-2^ s^-1^ respectively.

### 2.3 Statistical analysis

We used a non-rectangular hyperbola model (Thornley and Johnson, 1990; Thornley, 1998) in a multilevel Bayesian (MB) framework (Clark and Gelfand, 2006) to estimate model parameters and associated uncertainties. The MBLRC (Multilevel Bayesian Light Response Curve) model has three primary components: (1) the likelihood model which describes the likelihood of the observed net photosynthesis rate (A_N_), (2) the process model which describes the photosynthesis response to light (PAR) based on the non-rectangular hyperbola model and process uncertainty associated with random effects, and (3) the prior distributions for model parameters and precision terms. The posterior distribution of all model parameters was obtained by combining these three parts (Wikle, 2003).

**The likelihood model:** We assumed that the observations of net photosynthesis rate (A_N_) are normally distributed for each observation *i* (*i* = 1, 2,… *n*) around mean photosynthesis rate (*µ*) with precision τ (1/variance):


Eq. 1 $$A_{\text{N}[i]}\sim \text{Normal}({\text{μ}}_{\left[\text{i}\right]},\text{τ})$$

**Figure 2:**
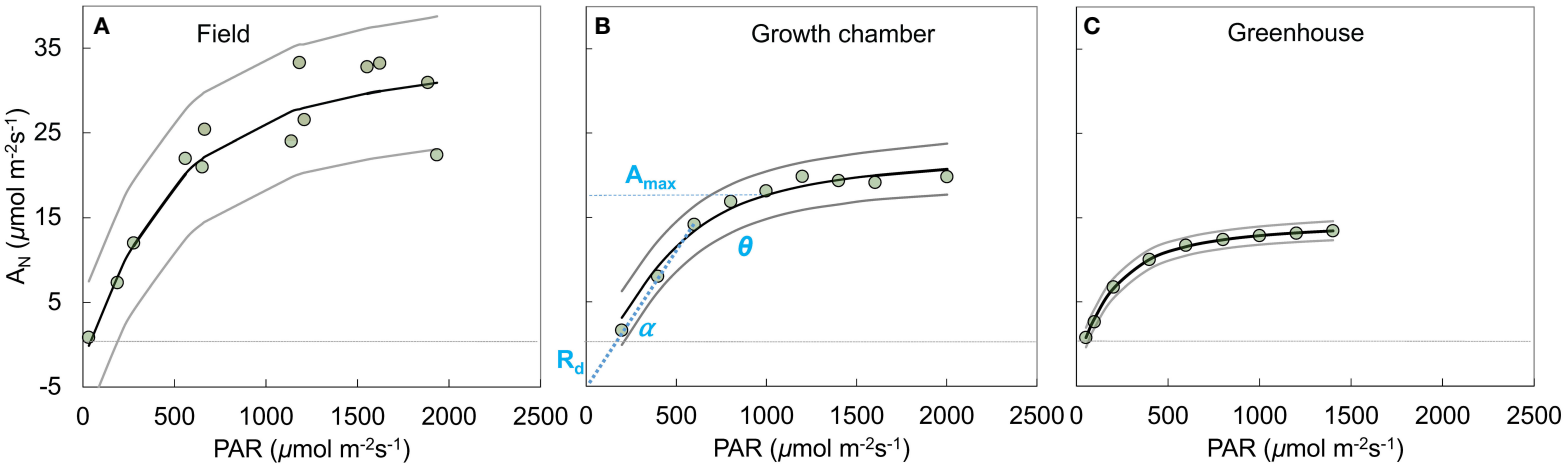
Example response curves (genotype = 310723) of photosynthesis (*A_N_*, *µ*mol CO_2_ m^-2^ s^-1^) to the photosynthetically active radiation (PAR, *µ*mol photon m^-2^ s^-1^) across field **(A)**, growth chamber **(B)**, and green house **(C)** conditions. The circles show the measurements, solid black (posterior mean) and gray (2.5% and 97.5% credible intervals) lines show predicted posterior distribution for each observation using the non-rectangular hyperbola model fit. Dotted horizontal line crosses the y axis at zero photosynthesis. Middle panel **(B)** shows the model parameters including maximum rate of photosynthesis (*A_max_*, *µ*mol CO_2_ m^-2^ s^-1^), mitochondrial respiration (*R_d_*, *µ*mol CO_2_ m^-2^ s^-1^), quantum yield of assimilation (*α*, initial slope of the curve, *µ*mol CO_2_ µmol photon^−1^), and a shape parameter (*θ*, unitless).

**The process model:** The process model describes the mean photosynthesis rate (*µ*) based on the non-rectangular hyperbola model as follows:


Eq. 2 $$\begin{array}{l} \mu_{\left[i\right]}\frac{a_{\left[Plant[i]\right]}\times PAR_{\left[i\right]}+Amax_{\left[Plant[i]\right]}-\sqrt{{\left(a_{\left[Plant[i]\right]}\times PAR_{\left[i\right]}+Amax_{\left[Plant[i]\right]}\right)}^{2}-4\theta_{\left[Plant[i]\right]}\times a_{\left[Plant[i]\right]}\times PAR_{\left[i\right]}+Amax_{\left[Plant[i]\right]}}}{2\theta_{\left[Plant[i]\right]}}\\ \;-Rd_{\left[Plant[i]\right]} \end{array}$$

where [Plant[i]] indicates plant-level parameters, *A_max_* is the maximum rate of assimilation (*µ*mol CO_2_ m^-2^ s^-1^), α is the apparent quantum efficiency (*µ*mol CO_2_ *µ*mol photon^−1^), *R_d_* is mitochondrial respiration (*µ*mol CO_2_ m^-2^ s^-1^), and *θ* (unitless) is the shape parameter for the curve (**Figure 2**).

**The parameter model:** Four unknown parameters of interest (*A_max_*, α, *R_d_*, and *θ*) are allowed to vary by each of the seven genotypes (*µ*.Parameter[s]), where s indicates the number of genotypes (s = 1,… *m*). For example, genotype-level parameters are described as:


Eq. 3 Parameter[s]~Normal(μ.Parameter,τ.Parameter)

where τ.Parameter is the precision term associated with the genotype-level mean (*µ*.Parameter) of the parameter of interest. *A_max_*, α, *R_d_*, and *θ* were given informative prior distributions with posterior means normally distributed around a mean reported for rice in published literature (e.g., Xu et al., 2019; Lv et al., 2020, see **Table 1** in appendix) and large ( ± 200%) variances associated with them.

**Figure 3:**
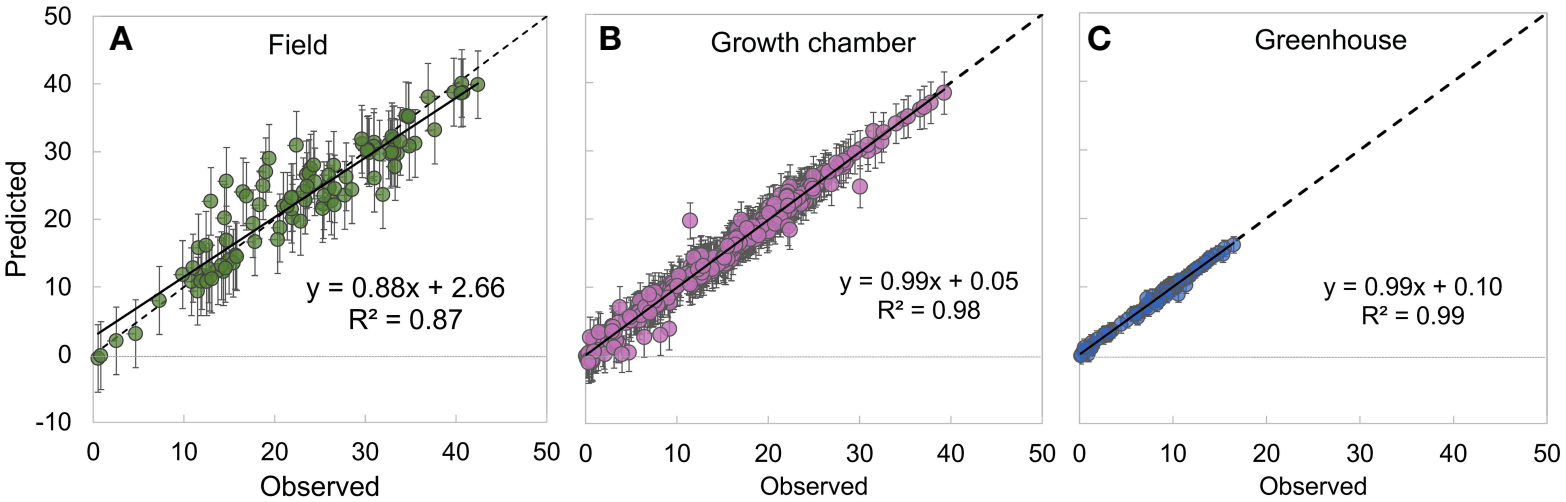
Relationship between observed and modeled values of the net photosynthesis rate (*A_N_*, *µ*mol CO_2_ m^-2^ s^-1^) showing the model goodness-of-fit across a field environment (*n* = 99) **(A)**, growth chamber environment (*n* = 366) **(B)**, and greenhouse environment (*n* = 86) **(C)**. Error bars represent 2.5% (bottom) and 97.5% (top) credible intervals. The dotted line represents 1:1 line and the solid black line represents the linear fit to the data.

The observed likelihood, process, and parameter models were combined to generate the posterior distributions of the unknown parameters (Wikle, 2003). The joint posterior was sampled by implementing the Markov Chain Monte Carlo (MCMC) algorithms (Robert and Casella, 2009) in the Bayesian statistical software package WinBUGS (Lunn et al., 2000) by running 3 parallel MCMC chains. Each MCMC chain was run for 10,000 iterations after convergence and the BGR diagnostic tool was used to evaluate convergence of the chains to the posterior distribution (Brooks and Gelman, 1998). The chains were thinned, due to autocorrelation in respiration parameter, every 10^th^ iteration to obtain an independent sample of 10,000 values per chain (total of 30,000 values) for each parameter from the joint posterior distribution. Model goodness-of-fit was evaluated by using Eq. 1 to generate modeled data for the observed photosynthesis values (Gelman et al., 2021) yielding posterior predictive distributions for each observation. The predicted means of photosynthesis with 95% credible intervals were compared with observed photosynthesis for evaluating the model goodness-of-fit (**Figure 3**).

The MBLRC model was fitted separately for three growing environments. For hypothesis testing (**µ*1* - **µ*2 = *0) differences in means across different genotypes, methods, and growing environments were calculated by conducting ANOVA and pairwise t-tests (Tukey HSD) on a representative (see **Figure S1** in **Supplementary Information**) random sample of 50 points for each parameter; random sample was taken to avoid the effect of large sample size on p-values.

## 3 Results

### 3.1 Model goodness-of-fit

The MBLRC model performed well in predicting observed A_N_ across all growing environments including field (R^2 ^= 0.87, **Figure 3A**), growth chamber (R^2 ^= 0.98, **Figure 3B**), and greenhouse (R^2 ^= 0.99, **Figure 3C**) environments.

### 3.2 Effect of measurement methods on phenotypic expression of photosynthesis

**Figure 4:**
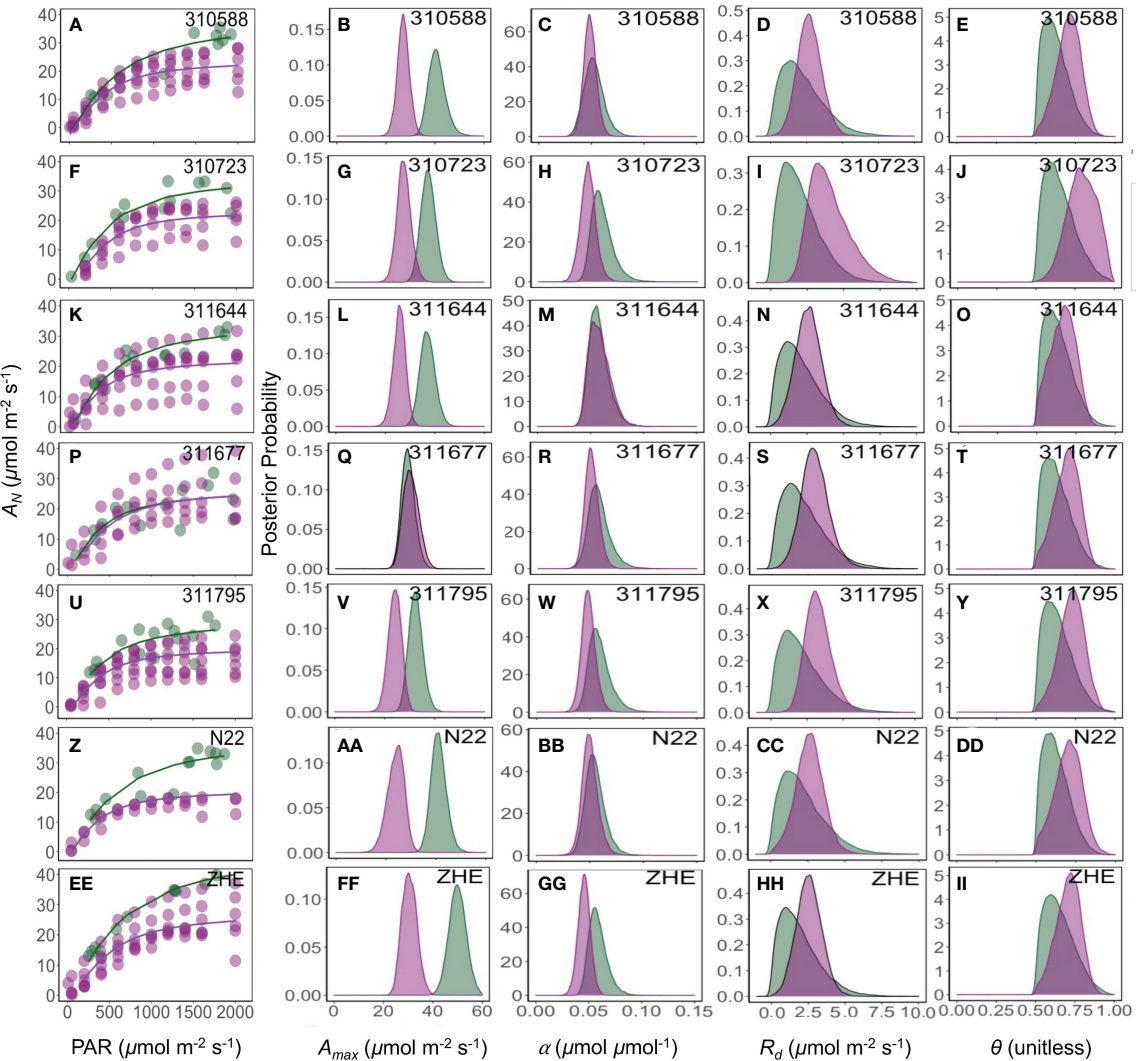
Response of photosynthesis (*A_N_*, *µ*mol CO_2_ m^-2^ s^-1^) to the photosynthetically active radiation (PAR, *µ*mol photon m^-2^ s^-1^) **(A, F, K, P, U, Z, EE)**. Green and pink lines are the fit of a non-rectangular hyperbola model for field and growth chamber plants, respectively. The posterior density distribution of photosynthesis model parameters *A_max_* (*µ*mol CO_2_ m^-2^ s^-1^) **(B, G, L, Q, V, AA, FF)**, α (*µ*mol CO_2_ *µ*mol photon^-1^) **(C, H, M, R, W, BB, GG)**, *R_d_* (*µ*mol CO_2_ m^-2^ s^-1^) **(D, I, N, S, X, CC, HH)**, and *θ* (unitless) **(E, J, O, T, Y, DD, II)** for seven rice (*Oryza sativa*) genotypes under field (green) and growth chamber (pink) conditions.

The maximum photosynthesis rate *(A_max_*) varied based on measurement method, and this variation was statistically different between the sequential and non-sequential methods across all genotypes (**Figure 4**) with exception of 311677 (**Figure 4Q**). While other parameters (α, *R_d_*, and *θ*) were generally similar between measurement methods with few exceptions (**Figure 4**). For example, *R_d_* was significantly lower in the non-sequential method in 310723 and *α* was significantly greater in the non-sequential method in 310723 and Zhe 733.

### 3.3 Effect of growing conditions on phenotypic expression of photosynthesis

**Figure 5:**
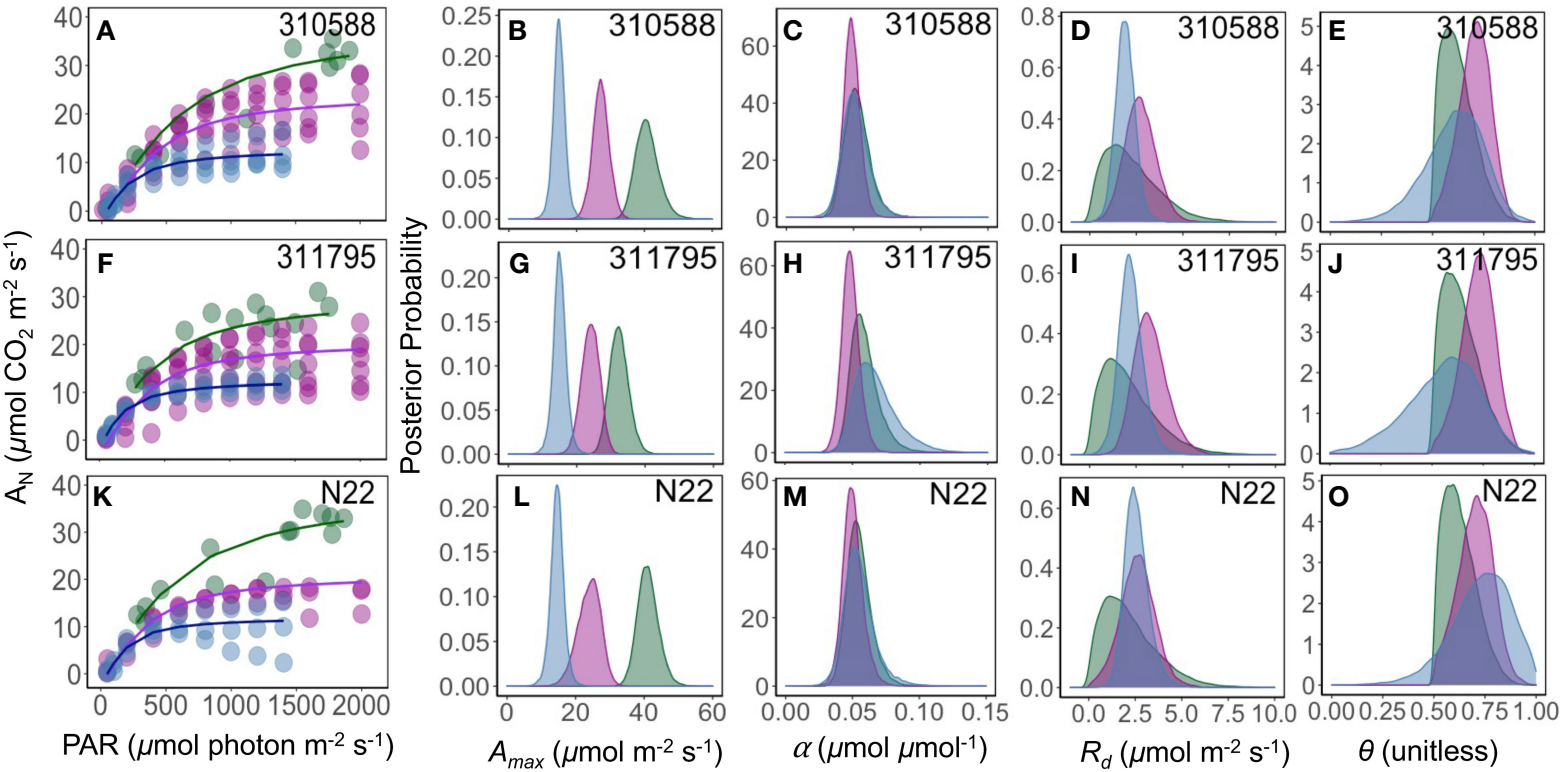
Response of photosynthesis (*A_N_*, *µ*mol CO_2_ m^-2^ s^-1^) to the photosynthetically active radiation (PAR, *µ*mol photon m^-2^ s^-1^) **(A, F, K)**. Green, pink, and blue lines are the fit of a non-rectangular hyperbola model for field, growth chamber, and greenhouse plants, respectively. The posterior density distribution of photosynthesis model parameter *A_max_* (*µ*mol CO_2_ m^-2^ s^-1^) **(B, G, L)**, α (*µ*mol CO_2_ *µ*mol photon^-1^) **(C, H, M)**, *R_d_* (*µ*mol CO_2_ m^-2^ s^-1^) **(D, I, N)**, and *θ* (unitless) **(E, J, O)** for three rice (*Oryza sativa*) genotypes under field (green), growth chamber (pink), and greenhouse (blue) conditions.

Consistent with the measurement method results, *α* remains largely conserved with no difference in parameter means, except one genotype (311795) where parameter (*α*) means were significantly different between greenhouse and growth chamber (**Figure 5**). The maximum photosynthesis rate (*A_max_*, *µ*mol CO_2_ m^-2^ s^-1^) was greater under field and growth chamber conditions as compared to the greenhouse conditions across all genotypes (**Figure 5**). Mitochondrial respiration (*R_d_*, *µ*mol CO_2_ m^-2^ s^-1^) and shape parameters (*θ*) were similar across all growing conditions. These measurements were conducted during the daytime, so photosynthesis measurements remained greater than zero at all light levels (**Figures 3**, **5**), and as a result *R_d_* and *θ* were correlated.

**Figure 6:**
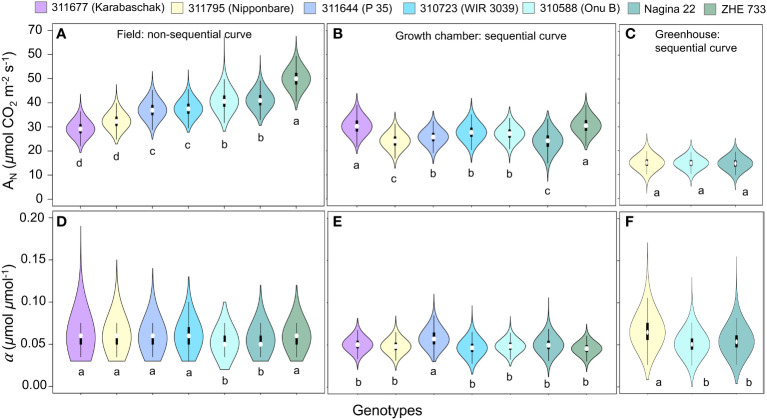
Comparison of photosynthesis model parameter *A_max_* (*µ*mol CO_2_ m^-2^ s^-1^) **(A–C)**, and α (*µ*mol CO_2_ *µ*mol photon^-1^) **(D–F)**, for rice (*Oryza sativa*) genotypes across different growing environments.

The pairwise comparison of model parameters across different genotypes showed that the quantum yield (*α*) remained generally similar across all genotypes, but field growing conditions were better suited for quantifying phenotypic difference in *A_max_* parameter as compared to growth chamber and greenhouse environments that showed minimal or no differences in model parameters (**Figure 6**). Under field conditions Zhe 733 showed the highest *A_max_* followed by Nagina 22 and 310588 (similar *A_max_*), 310723 and 311644 (similar *A_max_*), and lastly 311795 and 311677 at the lowest spectrum of *A_max_* values (**Figure 6A**).

## 4 Discussion

### 4.1 Effect of measurement methods on phenotypic expression of photosynthesis

Our results show that the non-sequential survey method (**Figure 2A**) captured greater variability compared to sequential method (**Figures 2B, C**). The two methods were very similar in their estimations of α, *R_d_*, and *θ*, but the non-sequential method resulted in a greater *A_max_* across nearly every genotype surveyed (**Figures 4**, **6**). However, these results could be the result of differences in pot grown plants acclimated to field conditions and plants grown in the field (**Figures 5**, **6**). Prior work has shown that plants grown under low light conditions and then moved to high light conditions before measurements increased their photosynthetic capacity (Walters, 2005; Athanasiou et al., 2010). Our results showed greater photosynthesis rate in growth chamber plants that were acclimated to field conditions before measurements as compared to greenhouse plants but increase in photosynthesis was not comparable to the plants grown under field conditions. To confirm that differences in parameter estimates between two methods were only due to the measurement methods, further experiments are needed. Overall, our results suggest that the non-sequential method is equally good or better when estimating the maximum photosynthetic rate of different genotypes to understand the interaction of genes, environment, and management under field conditions as it does not repeatedly measure the same sample, can be done at different days, and is less time consuming because samples are already acclimated to the surrounding environmental conditions.

### 4.2 Effect of growing conditions on phenotypic expression of photosynthesis

Light response curves play a vital role in understanding plant characteristics and quantifying photosynthetic acclimation under different conditions (Herrmann et al., 2019). Our results show that the maximum photosynthesis rate (*A_max_*) varied most significantly across different growing conditions. *A_max_* was greatest in the field plants across all genotypes (**Figure 5**, column 2, **Figure 6A**). Plants acclimate to the environment they are in (Walters, 2005; Dyson et al., 2015; Herrmann et al., 2019), and it has been well documented that plants grown at higher light intensities, such as those outside in the field, have greater *A_max_* than those at grown under lower light intensities, such as in a greenhouse (Ögren, 1993; Bailey et al., 2001; Walters, 2005; Perkins et al., 2006; Athanasiou et al., 2010; Du et al., 2020). Prior work has also shown that plants grown at low light and then moved to highlight before measurements increased their photosynthetic capacity (Walters, 2005; Athanasiou et al., 2010). Our results show that the growth chamber plants, which were grown under lower light intensities (600 *µ*mol m^-2^ s^-1^) and then moved to field conditions before measurements, often had *A_max_* values similar to field plants. The high variability seen in the light response curves of different growth chamber replicates may indicate that some leaves may have been shaded and not fully acclimated to the new light intensity, and thus had lower maximum photosynthesis rate (**Figures 4**, **5**, first column).

We expected a smaller shape parameter (*θ*) in field plants as *θ* is generally smaller in plants acclimated to high light (Ögren, 1993). Typically, *θ* ranges from 0.7 to 0.99 (*θ* = 1 is a Blackman curve and *θ* = 0 is a rectangular hyperbola (Ögren and Evans, 1993; Evans et al., 1993)). Ögren (1993) found that when grown under low light, algal cells, and willow (*Salix*) leaves had lower *A_max_* and higher *θ* than when grown at high light intensity, a finding that was only partially supported by our results. While our greenhouse plants had lower *A_max_* compared with field and growth chamber plants, *θ* was generally similar across all growing environments, except 311795 genotype (**Figure 5**).

Growth chamber plants showed greater variability across replicates within the same genotypes with some replicates showing curves similar to field conditions and some similar to greenhouse conditions (**Figure 5**). When plants, grown under controlled low light conditions, are allowed to acclimate outside for several days before measurements, photosynthesis measurements may be very similar to field plants. Field curves were different from those conducted on greenhouse plants, but the smallest curves of the growth chamber plants were similar to those of the greenhouse plants (**Figure 5**). Our results captured a gradient of growing conditions and plasticity of rice genotypes acclimated to those conditions.

The pairwise comparison of model parameters across different genotypes under field conditions showed that Zhe 733 had the highest *A_max_* followed by Nagina 22 and 310588 (similar *A_max_*), 310723 and 311644 (similar *A_max_*), and lastly 311795 and 311677 at the lowest spectrum of *A_max_* values (**Figure 6A**). Prior work has shown that Zhe 733 is sensitive to environmental stress (Moldenhauer et al., 2020), and our results showed that the *A_max_* almost doubled under field conditions as compared to the growth chamber, while 311677 remained the same across different light conditions (**Figures 6A, B**). All other genotypes showed little to moderate gain under field conditions compared to growth chamber (**Figures 6A, B**), but all genotypes showed significant increase in *A_max_* under field conditions compared to greenhouse conditions (**Figures 6A, C**).

Photosynthesis model parameters are a critical piece for understanding the gene and environmental interactions of different plant genotypes (Rascher et al., 2000). Photosynthetic parameters generally differ between field and lab experiments (Mishra et al., 2012; Tian-gen et al., 2017), and our results were consistent with the previous studies showing these differences. Field plants are exposed to a variety of light, temperature, and humidity conditions that cannot be easily replicated in a greenhouse. Additionally, greenhouse-grown plants may not have the nutrition, soil depth, etc. to meet their own growth demands. Thus, the photosynthetic parameters and physiological characteristics obtained from greenhouse plants should not be taken as representative of field-grown plants (Tian-gen et al., 2017), but the growth chamber or greenhouse grown plants acclimated to field conditions before measurements may be used for pot experiments.

## 5 Conclusions

Our results highlight the effect of measurement methods and growing conditions on photosynthesis model parameters and show that a non-sequential method used under field conditions can serve as a time efficient and equally valid tool for generating light response curves to understand gene and environment interactions. Additionally, our results show that the maximum photosynthesis rate *(A_max_)* is the parameter that varies the most in response to the growing conditions and measurement methods, while quantum yield of assimilation *(α)* is conserved across growing conditions and measurement methods. Our results suggest that measurement method and growing conditions should be carefully chosen to complement the goals of phenotyping experiments.

## Data availability statement

Data are available from the Dryad Digital Repository: DOI: 10.5061/dryad.6wwpzgn22, and model parameters can be found in the article/**Supplementary Material**.

## Author contributions

MR and KN designed this study. MR collected and analyzed data, KN developed and tested MBLRC model and conducted Bayesian analysis, MR and KN wrote the paper. AP provided rice seeds and resources to conduct greenhouse and field experiments including access to field plots, LP assisted in designing growth chamber experiments and provided resources to conduct growth chamber experiments. All authors contributed to the article and approved the submitted version.
