# Supplementary material for: Effects of measurement methods and growing conditions on phenotypic expression of photosynthesis in seven diverse rice genotypes
Source: Front Plant Sci. 2023 Sep 21;14:1106672. doi: 10.3389/fpls.2023.1106672 (PMC10551151; doi:10.3389/fpls.2023.1106672)

**Supplementary Information: Effects of Growing Conditions and Measurement Methods on Phenotypic Expression of Photosynthesis**

Megan Reavis^1^, Larry C. Purcell^2^, Andy Pereira^2^, and Kusum Naithani^1^*

^1^Department of Biological Sciences, University of Arkansas, Fayetteville, AR 72701

^2^Department of Crop, Soil, and Environmental Sciences, University of Arkansas, Fayetteville, AR 72701

| **Table S1.** Informed priors for the model parameters from literature. | | |
| --- | --- | --- |
| **Model Parameters** | **Priors from Literature** | |
|  | **Xu et al., 2019** | **LV et al., 2020** |
| *A_max_ ,* 𝜇mol CO_2_ m^-2^ s^-1^ | 26.225 | 35.920 |
| *α, µ*mol CO_2_ µmol photon^−1^ | 0.042 | 0.056 |
| *R_d_,* 𝜇mol CO_2_ m^-2^ s^-1^ | 0.594 | 0.730 |
| *Θ* | 0.750 | 0.684 |

**Table S2.** Summary statistics for genotype-level parameters in field including *A_max_* (maximum rate of photosynthesis), *Rd* (mitochondrial respiration), ⍺ (apparent quantum efficiency of photosynthesis), and θ (curvature shape parameter). [1] = 310588, [2] = 310723, [3] = 311644, [4] = 311677, [5] = 311795, [6] = Nagina 22, [7] = Zhe733. D is the sum of the squared deviation and sd is the standard deviation of modeled observations.

| **Node** | **Mean** | **SD** | **2.50%** | **Median** | **97.50%** | **Sample** |
| --- | --- | --- | --- | --- | --- | --- |
| D | 2669.0000 | 411.9000 | 1957.0000 | 2638.0000 | 3565.0000 | 29985 |
| sig.obs | 3.6880 | 0.2864 | 3.1790 | 3.6710 | 4.2980 | 29985 |
| **Genotype-level parameters** | | |  |  |  |  |
| sig.A_max_ | 7.2740 | 1.5640 | 4.2090 | 7.3350 | 9.8330 | 29985 |
| sig.Rd | 0.7548 | 0.7046 | 0.0230 | 0.5540 | 2.6620 | 29985 |
| sig.alpha | 0.0072 | 0.0065 | 0.0003 | 0.0056 | 0.0235 | 29985 |
| sig.theta | 0.0587 | 0.0560 | 0.0019 | 0.0438 | 0.1965 | 29985 |
| Amax[1] | 40.8200 | 3.4720 | 34.7900 | 40.5800 | 48.3900 | 29985 |
| Amax[2] | 37.5700 | 2.9750 | 31.9800 | 37.4900 | 43.6500 | 29985 |
| Amax[3] | 37.0400 | 3.0660 | 31.3200 | 36.9300 | 43.3800 | 29985 |
| Amax[4] | 29.3000 | 2.6870 | 24.4400 | 29.1500 | 35.0100 | 29985 |
| Amax[5] | 32.3900 | 2.7980 | 27.2700 | 32.2700 | 38.2500 | 29985 |
| Amax[6] | 41.1300 | 3.0860 | 35.5000 | 40.9700 | 47.6200 | 29985 |
| Amax[7] | 49.9900 | 3.5000 | 43.2400 | 49.9400 | 56.9500 | 29985 |
| Rd[1] | 2.2520 | 1.4790 | 0.2169 | 1.9810 | 5.7990 | 29985 |
| Rd[2] | 2.0250 | 1.3650 | 0.1673 | 1.7720 | 5.2870 | 29985 |
| Rd[3] | 2.0850 | 1.4070 | 0.1869 | 1.8120 | 5.4700 | 29985 |
| Rd[4] | 2.2120 | 1.4930 | 0.2007 | 1.9170 | 5.8290 | 29985 |
| Rd[5] | 2.1420 | 1.4820 | 0.1814 | 1.8480 | 5.7540 | 29985 |
| Rd[6] | 2.2050 | 1.5040 | 0.1927 | 1.9060 | 5.8110 | 29985 |
| Rd[7] | 1.9290 | 1.3620 | 0.1393 | 1.6540 | 5.2220 | 29985 |
| alpha[1] | 0.0524 | 0.0095 | 0.0357 | 0.0518 | 0.0733 | 29985 |
| alpha[2] | 0.0600 | 0.0102 | 0.0445 | 0.0584 | 0.0842 | 29985 |
| alpha[3] | 0.0580 | 0.0095 | 0.0431 | 0.0567 | 0.0801 | 29985 |
| alpha[4] | 0.0578 | 0.0106 | 0.0409 | 0.0564 | 0.0826 | 29985 |
| alpha[5] | 0.0592 | 0.0107 | 0.0430 | 0.0576 | 0.0845 | 29985 |
| alpha[6] | 0.0549 | 0.0091 | 0.0393 | 0.0540 | 0.0755 | 29985 |
| alpha[7] | 0.0587 | 0.0086 | 0.0451 | 0.0576 | 0.0786 | 29985 |
| theta[1] | 0.6255 | 0.0803 | 0.5101 | 0.6135 | 0.8093 | 29985 |
| theta[2] | 0.6519 | 0.0965 | 0.5137 | 0.6357 | 0.8751 | 29985 |
| theta[3] | 0.6352 | 0.0853 | 0.5119 | 0.6226 | 0.8280 | 29985 |
| theta[4] | 0.6339 | 0.0862 | 0.5107 | 0.6206 | 0.8319 | 29985 |
| theta[5] | 0.6408 | 0.0905 | 0.5118 | 0.6265 | 0.8494 | 29985 |
| theta[6] | 0.6267 | 0.0801 | 0.5099 | 0.6151 | 0.8086 | 29985 |
| theta[7] | 0.6526 | 0.0946 | 0.5150 | 0.6383 | 0.8676 | 29985 |

**Table S3.** Summary statistics for plant-level and genotype-level parameters in growth chamber including *A_max_* (maximum rate of photosynthesis), *Rd* (mitochondrial respiration), ⍺ (apparent quantum efficiency of photosynthesis), and θ (curvature shape parameter). [1] = 310588, [2] = 310723, [3] = 311644, [4] = 311677, [5] = 311795, [6] = Nagina 22, [7] = Zhe733. D is the sum of the squared deviation and sd is the standard deviation of modeled observations.

| **Node** | **Mean** | **SD** | **2.50%** | **Median** | **97.50%** | **Sample** |
| --- | --- | --- | --- | --- | --- | --- |
| D | 1298.0000 | 139.9000 | 1043.0000 | 1291.0000 | 1589.0000 | 29991 |
| sig.obs | 1.3320 | 0.0720 | 1.1960 | 1.3300 | 1.4770 | 29991 |
| **Genotype level parameters** | | |  |  |  |  |
| sig.Amax | 6.8850 | 0.9791 | 5.1710 | 6.8030 | 9.0210 | 29991 |
| sig.Rd | 1.9010 | 0.5556 | 0.9148 | 1.8680 | 3.0850 | 29991 |
| sig.alpha | 0.0124 | 0.0029 | 0.0073 | 0.0122 | 0.0186 | 29991 |
| sig.theta | 0.0975 | 0.0560 | 0.0060 | 0.0980 | 0.2092 | 29991 |
| mu.Amax[1] | 27.1600 | 2.4650 | 22.2700 | 27.1200 | 32.1100 | 29991 |
| mu.Amax[2] | 27.7500 | 2.8380 | 22.3600 | 27.6500 | 33.6200 | 29991 |
| mu.Amax[3] | 25.6600 | 2.5000 | 20.5300 | 25.7300 | 30.4100 | 29991 |
| mu.Amax[4] | 30.3600 | 3.1100 | 24.7600 | 30.2000 | 36.7100 | 29991 |
| mu.Amax[5] | 24.0400 | 2.6220 | 18.7700 | 24.0800 | 28.9900 | 29991 |
| mu.Amax[6] | 23.9100 | 3.3590 | 16.8200 | 24.1600 | 29.8700 | 29991 |
| mu.Amax[7] | 30.5400 | 2.9870 | 25.1200 | 30.4500 | 36.6100 | 29991 |
| mu.Rd[1] | 2.7090 | 0.8454 | 1.1170 | 2.6800 | 4.4420 | 29991 |
| mu.Rd[2] | 4.0500 | 1.3930 | 1.9920 | 3.8040 | 7.3360 | 29991 |
| mu.Rd[3] | 2.5850 | 0.8913 | 0.8731 | 2.5750 | 4.3860 | 29991 |
| mu.Rd[4] | 2.9850 | 0.9799 | 1.2090 | 2.9240 | 5.1120 | 29991 |
| mu.Rd[5] | 3.2890 | 0.8972 | 1.7430 | 3.2110 | 5.2550 | 29991 |
| mu.Rd[6] | 2.6350 | 0.9423 | 0.7572 | 2.6340 | 4.5180 | 29991 |
| mu.Rd[7] | 2.5520 | 0.8608 | 0.9190 | 2.5360 | 4.2840 | 29991 |
| mu.alpha[1] | 0.0487 | 0.0060 | 0.0372 | 0.0485 | 0.0609 | 29991 |
| mu.alpha[2] | 0.0467 | 0.0073 | 0.0327 | 0.0466 | 0.0620 | 29991 |
| mu.alpha[3] | 0.0582 | 0.0096 | 0.0428 | 0.0571 | 0.0792 | 29991 |
| mu.alpha[4] | 0.0508 | 0.0065 | 0.0389 | 0.0504 | 0.0648 | 29991 |
| mu.alpha[5] | 0.0485 | 0.0066 | 0.0363 | 0.0482 | 0.0624 | 29991 |
| mu.alpha[6] | 0.0503 | 0.0076 | 0.0365 | 0.0497 | 0.0668 | 29991 |
| mu.alpha[7] | 0.0456 | 0.0057 | 0.0342 | 0.0457 | 0.0569 | 29991 |
| mu.theta[1] | 0.7058 | 0.0763 | 0.5481 | 0.7094 | 0.8465 | 29991 |
| mu.theta[2] | 0.7927 | 0.0916 | 0.6081 | 0.7938 | 0.9533 | 29991 |
| mu.theta[3] | 0.6675 | 0.0782 | 0.5181 | 0.6709 | 0.8111 | 29991 |
| mu.theta[4] | 0.6961 | 0.0782 | 0.5338 | 0.7006 | 0.8372 | 29991 |
| mu.theta[5] | 0.7249 | 0.0801 | 0.5567 | 0.7283 | 0.8748 | 29991 |
| mu.theta[6] | 0.7033 | 0.0848 | 0.5318 | 0.7064 | 0.8645 | 29991 |
| mu.theta[7] | 0.7073 | 0.0771 | 0.5462 | 0.7119 | 0.8460 | 29991 |
| **Plant level parameters** | |  |  |  |  |  |
| Amax[1] | 27.9900 | 2.4760 | 23.1900 | 27.9800 | 32.8600 | 29991 |
| Amax[2] | 29.7900 | 1.8870 | 26.1700 | 29.7700 | 33.6400 | 29991 |
| Amax[3] | 20.1800 | 1.3640 | 17.7000 | 20.1200 | 23.0200 | 29991 |
| Amax[4] | 34.7300 | 2.3830 | 30.2300 | 34.6500 | 39.6100 | 29991 |
| Amax[5] | 31.5600 | 1.6380 | 28.5200 | 31.4700 | 34.9800 | 29991 |
| Amax[6] | 19.2500 | 1.7270 | 16.1000 | 19.1900 | 22.8600 | 29991 |
| Amax[7] | 30.9300 | 2.4550 | 26.4800 | 30.8100 | 36.0100 | 29991 |
| Amax[8] | 20.4700 | 2.3840 | 15.9900 | 20.4200 | 25.3200 | 29991 |
| Amax[9] | 33.2400 | 2.6680 | 28.2700 | 33.1800 | 38.6500 | 29991 |
| Amax[10] | 29.7400 | 2.7110 | 25.1100 | 29.5100 | 35.5000 | 29991 |
| Amax[11] | 26.8600 | 2.5670 | 22.0700 | 26.7800 | 32.1000 | 29991 |
| Amax[12] | 30.2400 | 2.2090 | 26.0800 | 30.1700 | 34.7000 | 29991 |
| Amax[13] | 18.2200 | 1.4460 | 15.5500 | 18.1600 | 21.1600 | 29991 |
| Amax[14] | 26.7400 | 1.3520 | 24.2500 | 26.6800 | 29.5700 | 29991 |
| Amax[15] | 26.8400 | 1.6630 | 23.7200 | 26.7900 | 30.2300 | 29991 |
| Amax[16] | 33.2800 | 1.2430 | 30.9700 | 33.2300 | 35.8800 | 29991 |
| Amax[17] | 13.6300 | 2.2130 | 9.5430 | 13.5500 | 18.1500 | 29991 |
| Amax[18] | 24.0800 | 2.2030 | 19.9400 | 24.0300 | 28.5300 | 29991 |
| Amax[19] | 29.6800 | 1.6880 | 26.5300 | 29.6300 | 33.1400 | 29991 |
| Amax[20] | 37.2600 | 2.8250 | 31.9100 | 37.1900 | 42.9300 | 29991 |
| Amax[21] | 44.2800 | 2.0430 | 40.6300 | 44.1700 | 48.6000 | 29991 |
| Amax[22] | 28.0400 | 3.3600 | 21.8600 | 27.9600 | 34.7600 | 29991 |
| Amax[23] | 25.4300 | 1.9280 | 21.8000 | 25.3700 | 29.3700 | 29991 |
| Amax[24] | 26.5900 | 1.8170 | 23.1700 | 26.5200 | 30.3000 | 29991 |
| Amax[25] | 20.3400 | 1.4330 | 17.7000 | 20.2800 | 23.2900 | 29991 |
| Amax[26] | 20.0500 | 2.1090 | 16.1400 | 20.0000 | 24.3700 | 29991 |
| Amax[27] | 29.9500 | 1.9340 | 26.3300 | 29.8900 | 33.9200 | 29991 |
| Amax[28] | 16.0300 | 1.4530 | 13.3000 | 15.9800 | 18.9600 | 29991 |
| Amax[29] | 18.4100 | 2.6670 | 13.5600 | 18.2800 | 23.9600 | 29991 |
| Amax[30] | 22.4600 | 1.5470 | 19.5600 | 22.4200 | 25.5800 | 29991 |
| Amax[31] | 20.9400 | 1.3990 | 18.4300 | 20.8600 | 23.8700 | 29991 |
| Amax[32] | 18.5700 | 1.5650 | 15.5900 | 18.5400 | 21.7100 | 29991 |
| Amax[33] | 29.1800 | 2.6930 | 24.3700 | 29.0100 | 34.9800 | 29991 |
| Amax[34] | 29.9400 | 2.3730 | 25.4700 | 29.9000 | 34.6900 | 29991 |
| Amax[35] | 30.9900 | 2.1550 | 27.1800 | 30.8600 | 35.6000 | 29991 |
| Amax[36] | 40.1600 | 2.8290 | 34.8600 | 40.0800 | 45.9600 | 29991 |
| Amax[37] | 41.7600 | 2.0880 | 38.0000 | 41.6200 | 46.2300 | 29991 |
| Amax[38] | 24.0000 | 2.4220 | 20.0300 | 23.8400 | 28.8200 | 29991 |
| Rd[1] | 4.7520 | 1.7730 | 1.5440 | 4.6810 | 8.4030 | 29991 |
| Rd[2] | 2.6280 | 1.0980 | 0.5787 | 2.5950 | 4.8540 | 29991 |
| Rd[3] | 1.5910 | 0.9373 | 0.1241 | 1.5040 | 3.6500 | 29991 |
| Rd[4] | 2.7870 | 1.4340 | 0.3551 | 2.6760 | 5.8660 | 29991 |
| Rd[5] | 0.7884 | 0.5749 | 0.0322 | 0.6795 | 2.1330 | 29991 |
| Rd[6] | 2.5440 | 1.2010 | 0.4029 | 2.4710 | 5.0530 | 29991 |
| Rd[7] | 4.3180 | 1.7510 | 1.1460 | 4.2170 | 7.9920 | 29991 |
| Rd[8] | 5.2730 | 1.9910 | 1.7320 | 5.1510 | 9.4590 | 29991 |
| Rd[9] | 4.1070 | 1.7330 | 0.9729 | 4.0040 | 7.7430 | 29991 |
| Rd[10] | 4.8970 | 1.7840 | 1.7220 | 4.7830 | 8.6470 | 29991 |
| Rd[11] | 5.6060 | 1.8920 | 2.2110 | 5.5070 | 9.5770 | 29991 |
| Rd[12] | 3.2980 | 1.6420 | 0.4796 | 3.1720 | 6.8030 | 29991 |
| Rd[13] | 2.4090 | 1.1340 | 0.3592 | 2.3550 | 4.7580 | 29991 |
| Rd[14] | 0.9011 | 0.6199 | 0.0454 | 0.8000 | 2.3240 | 29991 |
| Rd[15] | 2.1840 | 1.1190 | 0.2663 | 2.1110 | 4.5540 | 29991 |
| Rd[16] | 0.1807 | 0.1740 | 0.0044 | 0.1294 | 0.6400 | 29991 |
| Rd[17] | 4.9470 | 1.9410 | 1.3650 | 4.8820 | 8.8910 | 29991 |
| Rd[18] | 4.2590 | 1.8060 | 1.0230 | 4.1700 | 8.0120 | 29991 |
| Rd[19] | 1.9400 | 1.0340 | 0.2035 | 1.8660 | 4.1330 | 29991 |
| Rd[20] | 3.1930 | 1.5390 | 0.5173 | 3.0930 | 6.4810 | 29991 |
| Rd[21] | 0.2622 | 0.2469 | 0.0067 | 0.1890 | 0.9040 | 29991 |
| Rd[22] | 5.7280 | 2.3690 | 1.6060 | 5.5420 | 10.8300 | 29991 |
| Rd[23] | 3.4110 | 1.5200 | 0.6510 | 3.3300 | 6.6240 | 29991 |
| Rd[24] | 2.4620 | 1.0980 | 0.4371 | 2.4290 | 4.6890 | 29991 |
| Rd[25] | 2.1710 | 1.0670 | 0.2889 | 2.1290 | 4.4050 | 29991 |
| Rd[26] | 5.2060 | 1.7920 | 2.0020 | 5.1180 | 8.9980 | 29991 |
| Rd[27] | 2.6290 | 1.1100 | 0.5602 | 2.5900 | 4.8990 | 29991 |
| Rd[28] | 2.9010 | 1.1260 | 0.7601 | 2.8770 | 5.1790 | 29991 |
| Rd[29] | 5.8830 | 2.3450 | 1.8520 | 5.6860 | 10.9000 | 29991 |
| Rd[30] | 2.3540 | 1.1030 | 0.3640 | 2.3020 | 4.6580 | 29991 |
| Rd[31] | 1.3400 | 0.8810 | 0.0718 | 1.2160 | 3.3200 | 29991 |
| Rd[32] | 2.7640 | 1.1450 | 0.6268 | 2.7320 | 5.1030 | 29991 |
| Rd[33] | 2.1610 | 1.0660 | 0.3167 | 2.1000 | 4.4190 | 29991 |
| Rd[34] | 3.7720 | 1.6310 | 0.8632 | 3.6810 | 7.1850 | 29991 |
| Rd[35] | 1.5870 | 0.9447 | 0.1267 | 1.4910 | 3.6700 | 29991 |
| Rd[36] | 2.8200 | 1.4450 | 0.3831 | 2.7060 | 5.9140 | 29991 |
| Rd[37] | 0.2415 | 0.2293 | 0.0063 | 0.1738 | 0.8449 | 29991 |
| Rd[38] | 2.7900 | 1.1860 | 0.6481 | 2.7260 | 5.2620 | 29991 |
| alpha[1] | 0.0436 | 0.0088 | 0.0289 | 0.0429 | 0.0629 | 29991 |
| alpha[2] | 0.0512 | 0.0079 | 0.0376 | 0.0505 | 0.0684 | 29991 |
| alpha[3] | 0.0553 | 0.0098 | 0.0385 | 0.0544 | 0.0767 | 29991 |
| alpha[4] | 0.0483 | 0.0076 | 0.0357 | 0.0476 | 0.0650 | 29991 |
| alpha[5] | 0.0554 | 0.0069 | 0.0436 | 0.0548 | 0.0707 | 29991 |
| alpha[6] | 0.0347 | 0.0096 | 0.0198 | 0.0334 | 0.0569 | 29991 |
| alpha[7] | 0.0519 | 0.0094 | 0.0368 | 0.0508 | 0.0729 | 29991 |
| alpha[8] | 0.0377 | 0.0115 | 0.0197 | 0.0363 | 0.0636 | 29991 |
| alpha[9] | 0.0478 | 0.0088 | 0.0337 | 0.0468 | 0.0676 | 29991 |
| alpha[10] | 0.0433 | 0.0090 | 0.0301 | 0.0418 | 0.0644 | 29991 |
| alpha[11] | 0.0437 | 0.0095 | 0.0285 | 0.0424 | 0.0653 | 29991 |
| alpha[12] | 0.0559 | 0.0107 | 0.0382 | 0.0549 | 0.0796 | 29991 |
| alpha[13] | 0.0596 | 0.0126 | 0.0376 | 0.0586 | 0.0867 | 29991 |
| alpha[14] | 0.0632 | 0.0092 | 0.0474 | 0.0624 | 0.0834 | 29991 |
| alpha[15] | 0.0589 | 0.0105 | 0.0415 | 0.0579 | 0.0823 | 29991 |
| alpha[16] | 0.0869 | 0.0104 | 0.0688 | 0.0860 | 0.1099 | 29991 |
| alpha[17] | 0.0523 | 0.0153 | 0.0244 | 0.0519 | 0.0833 | 29991 |
| alpha[18] | 0.0451 | 0.0106 | 0.0275 | 0.0442 | 0.0681 | 29991 |
| alpha[19] | 0.0550 | 0.0083 | 0.0411 | 0.0542 | 0.0733 | 29991 |
| alpha[20] | 0.0514 | 0.0079 | 0.0380 | 0.0506 | 0.0689 | 29991 |
| alpha[21] | 0.0677 | 0.0066 | 0.0563 | 0.0672 | 0.0818 | 29991 |
| alpha[22] | 0.0378 | 0.0096 | 0.0233 | 0.0365 | 0.0595 | 29991 |
| alpha[23] | 0.0495 | 0.0094 | 0.0335 | 0.0486 | 0.0700 | 29991 |
| alpha[24] | 0.0548 | 0.0090 | 0.0394 | 0.0541 | 0.0745 | 29991 |
| alpha[25] | 0.0576 | 0.0103 | 0.0399 | 0.0566 | 0.0805 | 29991 |
| alpha[26] | 0.0461 | 0.0103 | 0.0286 | 0.0453 | 0.0684 | 29991 |
| alpha[27] | 0.0502 | 0.0080 | 0.0365 | 0.0495 | 0.0679 | 29991 |
| alpha[28] | 0.0457 | 0.0109 | 0.0273 | 0.0448 | 0.0695 | 29991 |
| alpha[29] | 0.0320 | 0.0113 | 0.0149 | 0.0304 | 0.0578 | 29991 |
| alpha[30] | 0.0544 | 0.0100 | 0.0375 | 0.0536 | 0.0763 | 29991 |
| alpha[31] | 0.0520 | 0.0097 | 0.0358 | 0.0511 | 0.0736 | 29991 |
| alpha[32] | 0.0459 | 0.0108 | 0.0274 | 0.0451 | 0.0693 | 29991 |
| alpha[33] | 0.0300 | 0.0055 | 0.0216 | 0.0293 | 0.0427 | 29991 |
| alpha[34] | 0.0441 | 0.0081 | 0.0305 | 0.0433 | 0.0618 | 29991 |
| alpha[35] | 0.0447 | 0.0070 | 0.0333 | 0.0439 | 0.0602 | 29991 |
| alpha[36] | 0.0454 | 0.0065 | 0.0348 | 0.0447 | 0.0601 | 29991 |
| alpha[37] | 0.0600 | 0.0058 | 0.0500 | 0.0596 | 0.0724 | 29991 |
| alpha[38] | 0.0343 | 0.0092 | 0.0216 | 0.0329 | 0.0541 | 29991 |
| theta[1] | 0.7563 | 0.1173 | 0.5125 | 0.7607 | 0.9608 | 29991 |
| theta[2] | 0.7207 | 0.1105 | 0.4761 | 0.7296 | 0.9125 | 29991 |
| theta[3] | 0.6891 | 0.1169 | 0.4274 | 0.6996 | 0.8847 | 29991 |
| theta[4] | 0.6941 | 0.1110 | 0.4441 | 0.7051 | 0.8798 | 29991 |
| theta[5] | 0.7057 | 0.1095 | 0.4628 | 0.7154 | 0.8926 | 29991 |
| theta[6] | 0.6422 | 0.1362 | 0.3177 | 0.6611 | 0.8580 | 29991 |
| theta[7] | 0.8545 | 0.0913 | 0.6373 | 0.8725 | 0.9789 | 29991 |
| theta[8] | 0.7466 | 0.1389 | 0.4264 | 0.7648 | 0.9559 | 29991 |
| theta[9] | 0.8087 | 0.1031 | 0.5672 | 0.8244 | 0.9583 | 29991 |
| theta[10] | 0.8978 | 0.0895 | 0.6667 | 0.9239 | 0.9960 | 29991 |
| theta[11] | 0.8165 | 0.1115 | 0.5514 | 0.8341 | 0.9748 | 29991 |
| theta[12] | 0.6806 | 0.1183 | 0.4358 | 0.6846 | 0.8980 | 29991 |
| theta[13] | 0.6611 | 0.1209 | 0.4023 | 0.6670 | 0.8806 | 29991 |
| theta[14] | 0.6348 | 0.1181 | 0.3744 | 0.6445 | 0.8369 | 29991 |
| theta[15] | 0.6397 | 0.1196 | 0.3715 | 0.6486 | 0.8483 | 29991 |
| theta[16] | 0.6279 | 0.1111 | 0.3796 | 0.6376 | 0.8152 | 29991 |
| theta[17] | 0.6441 | 0.1284 | 0.3618 | 0.6521 | 0.8722 | 29991 |
| theta[18] | 0.6582 | 0.1229 | 0.3840 | 0.6693 | 0.8669 | 29991 |
| theta[19] | 0.6531 | 0.1158 | 0.3945 | 0.6643 | 0.8461 | 29991 |
| theta[20] | 0.7030 | 0.1115 | 0.4684 | 0.7098 | 0.9003 | 29991 |
| theta[21] | 0.6416 | 0.1115 | 0.3857 | 0.6534 | 0.8216 | 29991 |
| theta[22] | 0.8019 | 0.1469 | 0.5181 | 0.8074 | 0.9961 | 29991 |
| theta[23] | 0.7189 | 0.1155 | 0.4568 | 0.7312 | 0.9082 | 29991 |
| theta[24] | 0.7502 | 0.1113 | 0.5042 | 0.7599 | 0.9345 | 29991 |
| theta[25] | 0.7440 | 0.1120 | 0.4963 | 0.7538 | 0.9326 | 29991 |
| theta[26] | 0.7623 | 0.1221 | 0.4971 | 0.7713 | 0.9686 | 29991 |
| theta[27] | 0.7194 | 0.1125 | 0.4654 | 0.7318 | 0.9016 | 29991 |
| theta[28] | 0.7006 | 0.1276 | 0.4097 | 0.7150 | 0.9086 | 29991 |
| theta[29] | 0.6843 | 0.1374 | 0.3665 | 0.7009 | 0.9065 | 29991 |
| theta[30] | 0.6935 | 0.1181 | 0.4363 | 0.7035 | 0.8948 | 29991 |
| theta[31] | 0.6720 | 0.1255 | 0.3918 | 0.6832 | 0.8816 | 29991 |
| theta[32] | 0.7186 | 0.1303 | 0.4456 | 0.7239 | 0.9559 | 29991 |
| theta[33] | 0.6733 | 0.1272 | 0.3821 | 0.6857 | 0.8891 | 29991 |
| theta[34] | 0.7040 | 0.1139 | 0.4574 | 0.7124 | 0.8999 | 29991 |
| theta[35] | 0.6265 | 0.1280 | 0.3256 | 0.6443 | 0.8284 | 29991 |
| theta[36] | 0.7087 | 0.1056 | 0.4804 | 0.7172 | 0.8891 | 29991 |
| theta[37] | 0.6764 | 0.1088 | 0.4309 | 0.6872 | 0.8563 | 29991 |
| theta[38] | 0.8446 | 0.1425 | 0.5496 | 0.8843 | 0.9986 | 29991 |

**Table S4.** Summary statistics for plant-level and genotype-level parameters in greenhouse including *A_max_* (maximum rate of photosynthesis), *Rd* (mitochondrial respiration), ⍺ (apparent quantum efficiency of photosynthesis), and θ (curvature shape parameter). [1] = 310588, [2] = 311795, [3] = Nagina 22. D is the sum of the squared deviation and sd is the standard deviation of modeled observations.

| **Node** | **Mean** | **SD** | **2.50%** | **Median** | **97.50%** | **Sample** |
| --- | --- | --- | --- | --- | --- | --- |
| D | 41.75 | 9.596 | 26.83 | 40.49 | 64.21 | 29991 |
| sig.obs | 0.49 | 0.056 | 0.40 | 0.49 | 0.62 | 29991 |
| **Genotype-level parameters** | | | |  |  |  |
| sig.Amax | 4.07 | 1.257 | 2.32 | 3.83 | 7.28 | 29991 |
| sig.Rd | 0.51 | 0.396 | 0.02 | 0.43 | 1.48 | 29991 |
| sig.alpha | 0.01 | 0.007 | 0.00 | 0.01 | 0.03 | 29991 |
| sig.theta | 0.24 | 0.106 | 0.07 | 0.22 | 0.49 | 29991 |
| mu.Amax[1] | 14.83 | 1.825 | 11.20 | 14.80 | 18.57 | 29991 |
| mu.Amax[2] | 15.00 | 1.976 | 11.08 | 14.96 | 19.05 | 29991 |
| mu.Amax[3] | 14.62 | 1.986 | 10.61 | 14.63 | 18.60 | 29991 |
| mu.Rd[1] | 1.91 | 0.522 | 0.92 | 1.90 | 2.97 | 29991 |
| mu.Rd[2] | 2.23 | 0.668 | 1.03 | 2.19 | 3.71 | 29991 |
| mu.Rd[3] | 2.58 | 0.658 | 1.48 | 2.52 | 4.06 | 29991 |
| mu.alpha[1] | 0.05 | 0.010 | 0.03 | 0.05 | 0.07 | 29991 |
| mu.alpha[2] | 0.07 | 0.016 | 0.04 | 0.06 | 0.11 | 29991 |
| mu.alpha[3] | 0.05 | 0.012 | 0.04 | 0.05 | 0.08 | 29991 |
| mu.theta[1] | 0.61 | 0.143 | 0.30 | 0.62 | 0.86 | 29991 |
| mu.theta[2] | 0.56 | 0.174 | 0.18 | 0.57 | 0.86 | 29991 |
| mu.theta[3] | 0.73 | 0.147 | 0.40 | 0.74 | 0.97 | 29991 |
| **Plant-level parameters** | | |  |  |  |  |
| **node** | **mean** | **sd** | **error** | **0.03** | **median** | **start** |
| Amax[1] | 12.59 | 0.827 | 10.92 | 12.61 | 14.15 | 29991 |
| Amax[2] | 12.43 | 0.781 | 10.99 | 12.40 | 14.07 | 29991 |
| Amax[3] | 21.37 | 1.428 | 18.76 | 21.32 | 24.27 | 29991 |
| Amax[4] | 13.03 | 0.778 | 11.55 | 13.02 | 14.60 | 29991 |
| Amax[5] | 17.19 | 1.045 | 15.25 | 17.15 | 19.32 | 29991 |
| Amax[6] | 15.00 | 0.905 | 13.30 | 14.98 | 16.82 | 29991 |
| Amax[7] | 13.54 | 0.863 | 11.89 | 13.52 | 15.28 | 29991 |
| Amax[8] | 18.55 | 1.001 | 16.69 | 18.51 | 20.60 | 29991 |
| Amax[9] | 12.67 | 0.869 | 11.23 | 12.58 | 14.66 | 29991 |
| Amax[10] | 11.85 | 1.266 | 9.91 | 11.67 | 14.75 | 29991 |
| Rd[1] | 1.66 | 0.611 | 0.40 | 1.67 | 2.84 | 29991 |
| Rd[2] | 1.80 | 0.590 | 0.65 | 1.79 | 2.99 | 29991 |
| Rd[3] | 2.05 | 0.536 | 1.07 | 2.03 | 3.19 | 29991 |
| Rd[4] | 1.93 | 0.574 | 0.86 | 1.90 | 3.14 | 29991 |
| Rd[5] | 2.34 | 0.708 | 1.11 | 2.28 | 3.91 | 29991 |
| Rd[6] | 2.25 | 0.699 | 0.99 | 2.20 | 3.78 | 29991 |
| Rd[7] | 2.10 | 0.702 | 0.79 | 2.07 | 3.61 | 29991 |
| Rd[8] | 2.29 | 0.653 | 1.08 | 2.27 | 3.67 | 29991 |
| Rd[9] | 2.61 | 0.647 | 1.51 | 2.55 | 4.06 | 29991 |
| Rd[10] | 3.05 | 0.886 | 1.68 | 2.92 | 5.12 | 29991 |
| alpha[1] | 0.04 | 0.010 | 0.02 | 0.04 | 0.06 | 29991 |
| alpha[2] | 0.06 | 0.012 | 0.04 | 0.06 | 0.09 | 29991 |
| alpha[3] | 0.04 | 0.007 | 0.03 | 0.04 | 0.06 | 29991 |
| alpha[4] | 0.06 | 0.012 | 0.04 | 0.06 | 0.09 | 29991 |
| alpha[5] | 0.07 | 0.015 | 0.04 | 0.06 | 0.10 | 29991 |
| alpha[6] | 0.07 | 0.016 | 0.05 | 0.07 | 0.11 | 29991 |
| alpha[7] | 0.07 | 0.017 | 0.05 | 0.07 | 0.11 | 29991 |
| alpha[8] | 0.06 | 0.011 | 0.05 | 0.06 | 0.09 | 29991 |
| alpha[9] | 0.05 | 0.012 | 0.04 | 0.05 | 0.08 | 29991 |
| alpha[10] | 0.04 | 0.011 | 0.03 | 0.04 | 0.07 | 29991 |
| theta[1] | 0.35 | 0.197 | 0.02 | 0.35 | 0.73 | 29991 |
| theta[2] | 0.86 | 0.112 | 0.56 | 0.89 | 0.99 | 29991 |
| theta[3] | 0.58 | 0.170 | 0.19 | 0.61 | 0.85 | 29991 |
| theta[4] | 0.58 | 0.172 | 0.18 | 0.60 | 0.85 | 29991 |
| theta[5] | 0.55 | 0.181 | 0.14 | 0.58 | 0.84 | 29991 |
| theta[6] | 0.50 | 0.184 | 0.10 | 0.52 | 0.80 | 29991 |
| theta[7] | 0.51 | 0.185 | 0.11 | 0.53 | 0.82 | 29991 |
| theta[8] | 0.62 | 0.146 | 0.27 | 0.64 | 0.84 | 29991 |
| theta[9] | 0.88 | 0.105 | 0.60 | 0.91 | 0.99 | 29991 |
| theta[10] | 0.89 | 0.140 | 0.48 | 0.94 | 1.00 | 29991 |

**Figure S1.** Comparison of the full distribution (n = 30,000) and the representative random sample (n = 50) for model parameters *A_max_* (a,d,g), *𝛼* (b,e,h), and *R_d_* (c,f,i) across field (F: a,b,c), growth chamber (GC: d,e,f), and green house (GH: g,h,i) conditions.


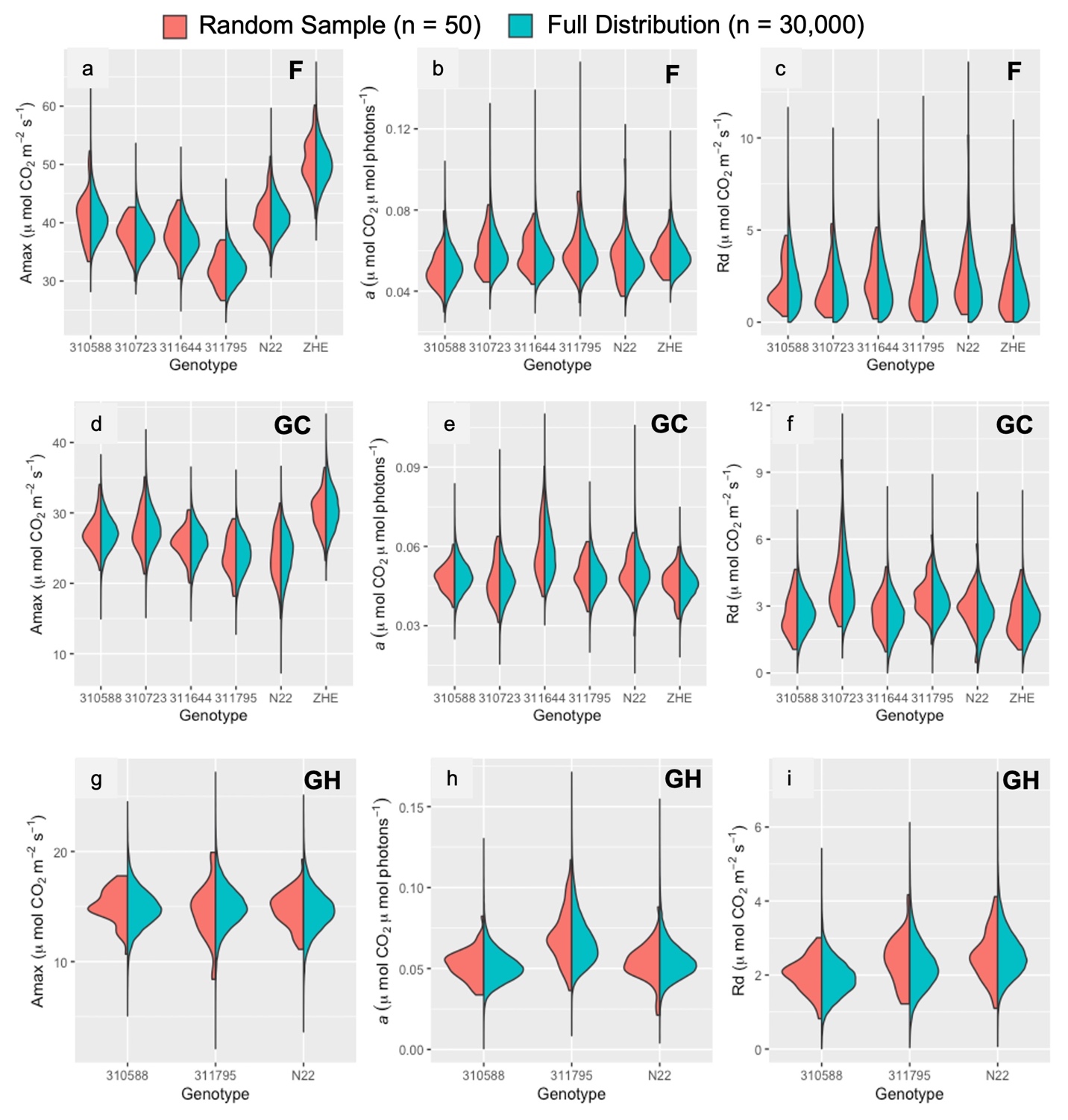

Supplement: Supplementary file 1 [file DataSheet_1.docx]
